# *SULT1A1 *rs9282861 polymorphism-a potential modifier of efficacy of the systemic adjuvant therapy in breast cancer?

**DOI:** 10.1186/1471-2407-12-257

**Published:** 2012-06-18

**Authors:** Maria Tengström, Arto Mannermaa, Veli-Matti Kosma, Ari Hirvonen, Vesa Kataja

**Affiliations:** 1 Cancer Center, Kuopio University Hospital, P.O.BOX 1777, 70211 Kuopio, Finland; 2 Department of Oncology, Vaasa Central Hospital, Hietalahdenkatu 2-4, 65130 Vaasa, Finland; 3 Institute of Clinical Medicine, Pathology; Biocenter Kuopio; Cancer Center of Eastern Finland, University of Eastern Finland, Yliopistonranta 1C, 70210 Kuopio, Finland; 4 Imaging Center, Pathology, Kuopio University Hospital, P.O.BOX 1777, 70211 Kuopio, Finland; 5 Finnish Institute of Occupational Health, Topeliuksenkatu 41 a A, 00250 Helsinki, Finland

## Abstract

**Background:**

Sulfotransferase 1A1 (SULT1A1) participates in the elimination of 4-hydroxy-tamoxifen (4-OH-TAM), which is one of the major active metabolites of tamoxifen (TAM). Homozygous *SULT1A1 *variant allele genotype has been associated with lower catalytic activity and thermostability of the enzyme. Previous clinical studies suggest that the *SULT1A1 *rs9282861 polymorphism may influence the survival of breast cancer patients treated with TAM in the adjuvant setting. We investigated the effect of rs9282861 genotypes on the survival of Finnish breast cancer patients treated with adjuvant chemotherapy or TAM.

**Methods:**

The rs9282861 genotypes of 412 Finnish breast cancer patients with early breast cancer were identified by using PCR-RFLP method. Seventy six patients were treated with adjuvant cyclophosphamide based chemotherapy only, 65 patients received adjuvant TAM, and four patients were treated with both adjuvant chemotherapy and TAM. Overall long-term survival (OS), breast cancer specific survival (BCSS), and relapse-free survival (RFS) by rs9282861 genotypes were evaluated by the Kaplan-Meier method and Cox regression analysis.

**Results:**

The multivariate analysis of 145 patients receiving either adjuvant TAM or chemotherapy showed a statistically significantly improved OS in patients with the rs9282861 homozygous variant AA genotype (hazard ratio [HR] = 0.50, 95% confidence interval [CI] = 0.29-0.88, *P *= 0.015). In the separate analyses of patients receiving only chemotherapy or adjuvant TAM, there were no statistically significant differences in survival.

**Conclusions:**

In this prospective study, we observed a previously unreported association between the *SULT1A1 *rs9282861 genotype and OS of breast cancer patients treated with adjuvant chemotherapy or TAM. This novel finding suggests that the rs9282861 polymorphism modifies the long-term clinical outcome of patients receiving adjuvant TAM or chemotherapy.

## Background

Tamoxifen (TAM) has been used for the treatment of oestrogen-receptor-positive breast cancer for three decades and still has its place in the treatment of both early and metastatic breast cancer. In the adjuvant setting it is the preferred endocrine therapy in premenopausal women and an acceptable option in postmenopausal women, especially in the group with low risk of relapse [1]. In early stage breast cancer, TAM reduces the 15-year risks of breast cancer recurrence and death by about a third [2]. Even though the benefit of adjuvant TAM persists for years, some patients will eventually relapse and die of breast cancer [2]. In addition to causing hot flushes TAM increases the risk of endometrial cancer and thromboembolic complications [3,4].

The most important metabolites of TAM in terms of therapeutic efficacy are 4-hydroxy-TAM (4-OH-TAM) and 4-OH-N-desmethyl-TAM (endoxifen) [5]. The detoxification of 4-OH-TAM is catalyzed by the phase II enzymes human sulfotransferase 1A1 (SULT1A1) and uridine diphosphate glucuronosyltransferase isoform 2B15 (UGT2B15) [6]. SULT1A1 is a member of the sulfotransferase family, which has the capability to sulphate phenolic and steroid compounds. A G683A base substitution (rs9282861) in exon 7 of SULT1A1 results in an Arg213His amino acid change with functional consequences; the variant A allele encodes an enzyme with lower catalytic activity and thermostability compared with the wild-type G allele [7]. The impact of *SULT1A1 *rs9282861 genotype on the risk of breast cancer and response to TAM therapy has been reported in several studies; the variant AA genotype has been associated both with poorer overall survival (OS) [8] and with no effect on OS [9,10], whereas patients with the homozygous wild-type GG genotype have been reported to have a tendency towards improved distant recurrence-free survival (RFS) [11].

In the 1970s Bonadonna et al. [12] presented the adjuvant chemotherapy regimen of cyclophosphamide (CPA), methotrexate, and 5-fluorouracil (CMF). This has been shown to significantly decrease the relative risk of relapse and death compared with no systemic treatment [13]. Newer agents such as anthracyclines and taxanes have further improved the survival of breast cancer patients [2,14]. CPA containing combinations are standard therapies in the adjuvant treatment of breast cancer [1,15]. The intravenous (i.v.) CMF (CPA 500 mg/m^2^, methotrexate 40 mg/m^2^, 5-fluorouracil 500 mg/m^2^) is a modification of the classical CMF, and it has been used in Finland as a standard adjuvant treatment, especially in the late 1980s and early 1990s.

To date, there are no published data on the effect of the *SULT1A1 *rs9282861 single nucleotide polymorphism (SNP) on the outcome of adjuvant chemotherapy or the long-term survival of breast cancer patients. However, there is evidence that SNPs of the genes coding for drug-metabolising enzymes may influence the outcome of chemotherapy. CPA is a pro-drug that is converted into the active cytotoxic alkylating phosphoramide mustard through the formation of 4-hydroxy-CPA (4-OHCPA) and its tautomer aldophosphamide [16]. SNPs of the cytochrome P450 (CYP) genes that are involved in the bioactivation of CPA, i.e., *CYP2B6, CYP2C9, CYP2C19 *and *CYP3A4/5*, may affect CPA pharmacokinetics [17-19]. Moreover, there is some evidence that polymorphisms of detoxifying glutathione-S-transferases (GSTs) may have an influence on the outcome of CPA treatment; homozygous variant GSTA1*B/*B genotype was related to reduced mortality during the first 5 years after diagnosis of breast cancer [20] and the low activity associated *GSTP1 Val105Val *genotype has been reported to confer to better survival [21].

In this prospective study, our aim was to determine whether *SULT1A1 *rs9282861 SNP influences the long-term survival of breast cancer patients receiving adjuvant chemotherapy or TAM.

## Methods

### Study population

The Kuopio Breast Cancer Project is a prospective case-control study that was conducted in 1990-1995 and was approved by the Kuopio University Hospital Board of Research Ethics. Briefly, women who were referred to Kuopio University Hospital due to breast symptoms were invited to take part in the study at their first visit to the hospital. The subjects provided written informed consent for the study. Extensive data regarding medical history, family history of breast cancer, socioeconomic background, alcohol use, and cigarette smoking were collected from the patients.

Altogether 520 women out of 1,919 were eventually diagnosed to have breast cancer. All patients were ethnic Finns. Hospital registries were used to collect information concerning clinicopathological features of the breast cancer, type and duration of treatment, and follow-up. For this particular study, patients who had carcinoma in situ, metastatic disease at presentation, unknown nodal status, prior history of breast cancer or refused surgery were excluded (n = 78). The *SULT1A1 *rs9282861 genotype data were available for 412 eligible patients.

There were 84 patients who received adjuvant TAM but no chemotherapy. Of these, five women were excluded because of the very short duration of TAM treatment, namely less than three months, and 14 patients were further excluded due to negative or unknown estrogen receptor (ER) status. Thus, 65 TAM treated patients were ultimately included in the planned analyses. In this group, the median duration of adjuvant TAM therapy was 36 months (range 3-75). Forty seven patients received a daily dosage of 20 mg TAM and 18 patients received a daily dosage of 40 mg TAM.

The rs9282861 genotype data were available for 76 patients who were treated with chemotherapy as their only systemic adjuvant treatment. Seventy patients were treated with CMF, whereas six patients received CNF (CPA 500 mg/m^2^, mitoxantrone 10 mg/m^2 ^and 5-fluorouracil 500 mg/m^2^). The median number of cycles was six (range 2-6). Four patients were treated with both adjuvant TAM and chemotherapy.

Data on breast cancer risk associated with various genetic polymorphisms in this study population have previously been reported by Mitrunen, Sillanpää and Hartikainen, and coworkers [22-24].

### Genotyping

One hundred ng of the DNA were used as a template in the genotyping analyses using a PCR-RFLP based method as reported by Engelke et al. [25]. Samples with known genotypes and nontemplate samples were used as positive and negative internal controls, respectively. Duplicates of 10% of the samples were blindly analyzed for quality control with fully concordant results.

### Statistical evaluation

The data on survival and causes of death were obtained from hospital registries which utilize the national Population Registry. The cause of death was classified either as caused by breast cancer or not caused by breast cancer. OS and breast cancer specific survival (BCSS) were calculated from the date of diagnosis to the last follow-up date or date of death. RFS was recorded from time of diagnosis to time of first relapse (local relapse, contra lateral breast cancer or metastatic disease) or the end of follow-up. The causes of death are specified according to the *SULT1A1 *rs9282861 genotype in the Additional file 1: Table S1, Table S2 and Table S3.

Statistical analyses were conducted using SPSS version 17.0. The impact of *SULT1A1 *rs9282861 genotype on RFS, BCSS, and OS were first calculated by using univariate Kaplan-Meier survival analysis and the significance of the differences detected between groups was assessed by the log-rank test. The survival was estimated for the homozygous carriers of the G allele compared with the carriers of the variant A allele, as well as for homozygous carriers of the variant allele compared with the carriers of the wild-type allele.

The *P*-values, and the hazard ratios (HRs) and their 95% confidence intervals (CIs) were calculated using Cox proportional hazards models adjusted for potential confounders. The *P*-value ≤ 0.05 was considered to be statistically significant.

## Results

### Allele and genotype distribution in relation to subject characteristics

Data on the patient characteristics and genotypes of the whole study population are presented in Table 1. The median age of the patients at the time of diagnosis was 56 years (range 23-91 years). The median follow-up time at the cut-off in February 2011 for the total study population was 11.9 years (range 0.1-20.4 years).

**Table 1:** Distribution of the *SULT1A1 *rs9282861 genotypes in relation to the subject characteristics^§^

		rs9282861 genotype		
		
Characteristics	All subjects (n = 412)	GG (n = 121)	AG (n = 194)	AA (n = 97)
Age at diagnosis, years				
≤ 39	37 (9.0)	14 (37.8)	15 (40.5)	8 (21.6)
40-49	98 (23.8)	34 (34.7)	40 (40.8)	24 (24.5)
50-59	104 (25.2)	29 (27.9)	51 (49.0)	24 (23.1)
60-69	65 (15.8)	16 (24.6)	36 (55.4)	13 (20.0)
≥ 70	108 (26.2)	28 (25.9)	52 (48.1)	28 (25.9)
T stage (UICC)^†^				
1	215 (52.2)	59 (27.4)	101 (47.0)	55 (25.6)
2	164 (39.8)	56 (34.1)	73 (44.5)	35 (21.3)
3	22 (5.3)	3 (13.6)	15 (68.2)	4 (18.2)
4	11 (2.7)	3 (27.3)	5 (45.5)	3 (27.3)
Nodal status (UICC)				
N-	247 (60.0)	67 (27.1)	117 (47.4)	63 (25.5)
N+	165 (40.0)	54 (32.7)	77 (46.7)	34 (20.6)
Stage (UICC)				
I	165 (40.0)	46 (27.9)	79 (47.9)	40 (24.2)
II	210 (51.0)	68 (32.4)	92 (43.8)	50 (23.8)
III	37 (9.0)	7 (18.9)	23 (62.2)	7 (18.9)
Oestrogen receptor status				
Positive	310 (75.2)	88 (28.4)	146 (47.1)	76 (24.5)
Negative	89 (21.6)	28 (31.5)	45 (50.6)	16 (18.0)
Unknown	13 (3.2)	5 (38.5)	3 (23.1)	5 (38.5)
Progesterone receptor status				
Positive	247 (60.0)	71 (28.7)	113 (45.7)	63 (25.5)
Negative	150 (36.4)	45 (30.0)	76 (50.7)	29 (19.3)
Unknown	15 (3.6)	5 (33.3)	5 (33.3)	5 (33.3)
Morphological type				
Ductal	268 (65.0)	75 (28.0)	132 (49.3)	61 (22.8)
Lobular	70 (17.0)	28 (40.0)	27 (38.6)	15 (21.4)
Other types	74 (18.0)	18 (24.3)	35 (47.3)	21 (28.4)
Adjuvant treatment				
Radiotherapy	243 (59.0)	74 (30.5)	110 (45.3)	59 (24.3)
Endocrine therapy	106 (25.7)	28 (26.4)	56 (52.8)	22 (20.8)
Tamoxifen	88 (21.4)	23 (26.1)	45 (51.1)	20 (22.7)
Toremifen	17 (4.1)	5 (29.4)	11 (64.7)	1 (5.9)
Chemotherapy	80 (19.4)	34 (42.5)	31 (38.8)	15 (18.8)
Vital Status				
Alive	184 (44.7)	50 (27.2)	88 (47.8)	46 (25.0)
No recurrence	159 (38.6)	41 (25.8)	79 (49.7)	39 (24.5)
Recurrence of breast cancer	25 (6.1)	9 (36.0)	9 (36.0)	7 (28.0)
Death	228 (55.3)	71 (31.1)	106 (46.5)	51 (22.4)
Breast cancer caused death	112 (27.2)	40 (35.7)	49 (43.8)	23 (20.5)
Death with other reasons	116 (28.2)	31 (26.7)	57 (49.1)	28 (24.1)

^§^Values in the table are number of patients (%).

^†^UICC = International Union Against Cancer, TNM Classification of Malignant Tumours, Fourth, Fully Revised Edition

The rs9282861 genotypes were in Hardy-Weinberg equilibrium in the whole study population. The wild-type G allele frequency was 53% in the whole study population (n = 412), 46% in the TAM-treated group (n = 65), and 61% in the chemotherapy treated group (n = 76). In the TAM-treated group the frequencies of the rs9282861 GG, AG and AA genotypes were 20.0%, 52.3% and 27.7%, respectively. In the group of chemotherapy treated patients the respective genotype frequencies were 40.8%, 40.8% and 18.4%.

### Rs9282861 SNP and survival of patients receiving adjuvant chemotherapy

**Figure 1:**
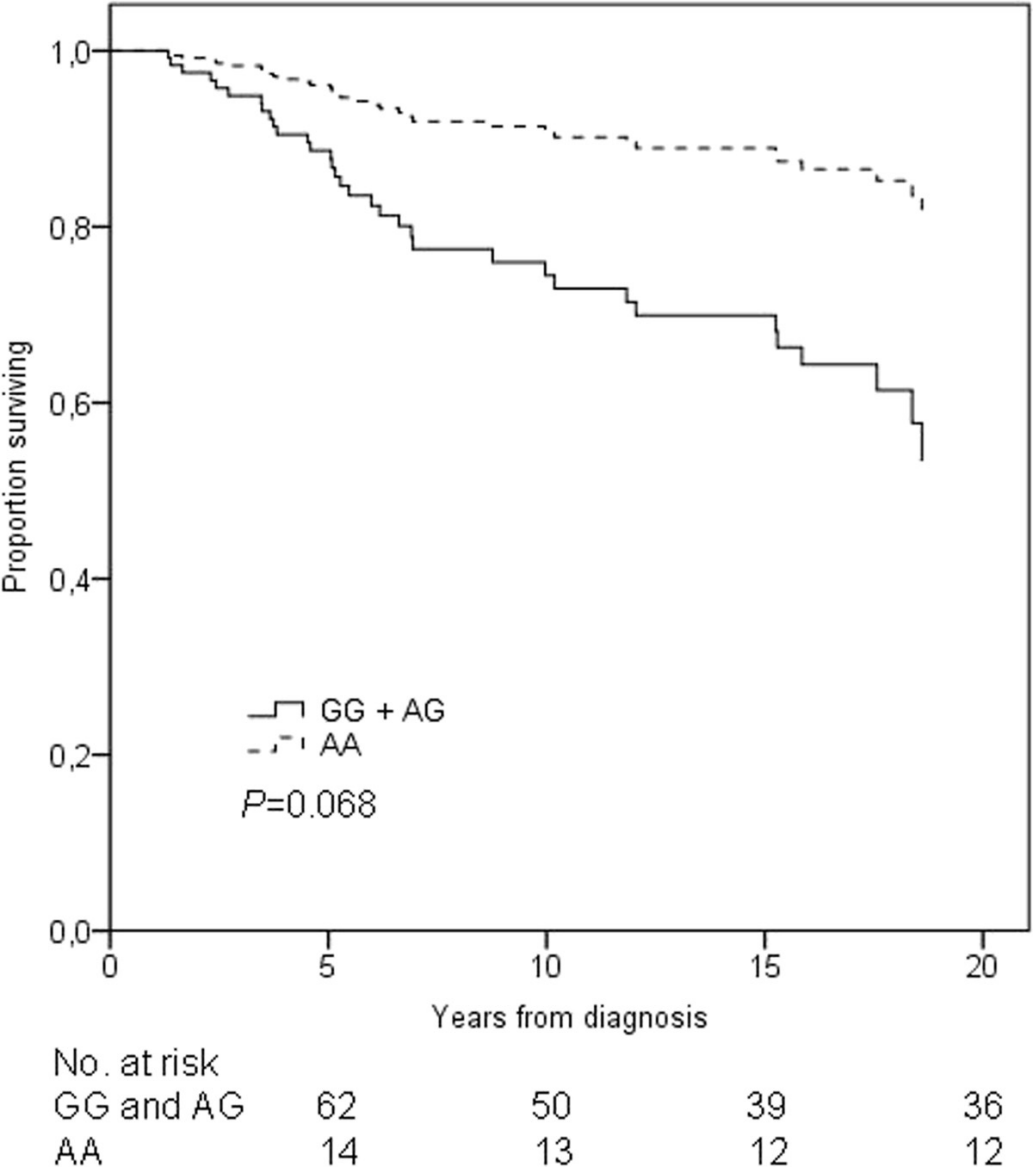
***SULT1A1 *rs9282861 genotype and overall survival of breast cancer patients receiving adjuvant chemotherapy**. The black solid line represents patients homozygous or heterozygous for the rs9282861 G allele and the black dotted line represents patients homozygous for the rs9282861 A allele. Adjustments were made for stage, age, radiation therapy, and hormone receptor status.

In the Kaplan-Meier OS analysis the *SULT1A1 *rs9282861 homozygous variant AA genotype emerged as a statistically significant favourable genotype compared with other genotypes (log-rank *P *= 0.045). A similar although statistically insignificant pattern was seen in BCSS (log-rank *P *= 0.075) (Additional file 3: Figure S2). In the recessive model after adjusting for age, stage of disease at diagnosis, adjuvant radiation therapy and oestrogen and progesterone receptor status, Cox regression analysis showed no statistically significant differences in OS (HR = 0.33, 95% CI = 0.10-1.09, *P *= 0.068) (Figure 1, Table 2), neither did the BCSS differ significantly (HR = 0.36, 95% CI = 0.11-1.20, *P *= 0.095) (data not shown). All three deaths among carriers of the homozygous variant genotype were caused by breast cancer, whereas among the carriers of the GG and AG genotypes 29 patients died of breast cancer and only three deaths were due to other causes. In contrast, RFS did not differ significantly between the different rs9282861 genotype groups (log-rank *P *= 0.17).

**Table 2:** Associations between the *SULT1A1 *rs9282861 genotypes, adjuvant treatment and overall survival (OS)

Cases	n	HR	95% CI	*P*^§^	median OS (years)
chemotherapy only	76				13.0

GG or AG	62	1^†^			11.0
AA	14	0.33	0.10 to 1.09	0.068	16.0

TAM only	65				9.2

GG or AG	47	1^‡^			8.2
AA	18	0.53	0.27 to 1.08	0.079	11.9

chemotherapy/TAM^¥^	145				10.0

GG or AG	112	1^†^			9.1
AA	33	0.50	0.29 to 0.88	0.015	13.7

Abbreviations: TAM = tamoxifen

*^§^P *was based on Cox proportional analysis

^†^Reference value; HRs adjusted for age and stage at diagnosis, radiation therapy, and hormone receptor status

^‡^Reference value; HRs adjusted for age and stage at diagnosis, and radiation therapy

^¥^Four patients received chemotherapy and TAM

### Rs9282861 SNP and survival of patients receiving adjuvant TAM

**Figure 2:**
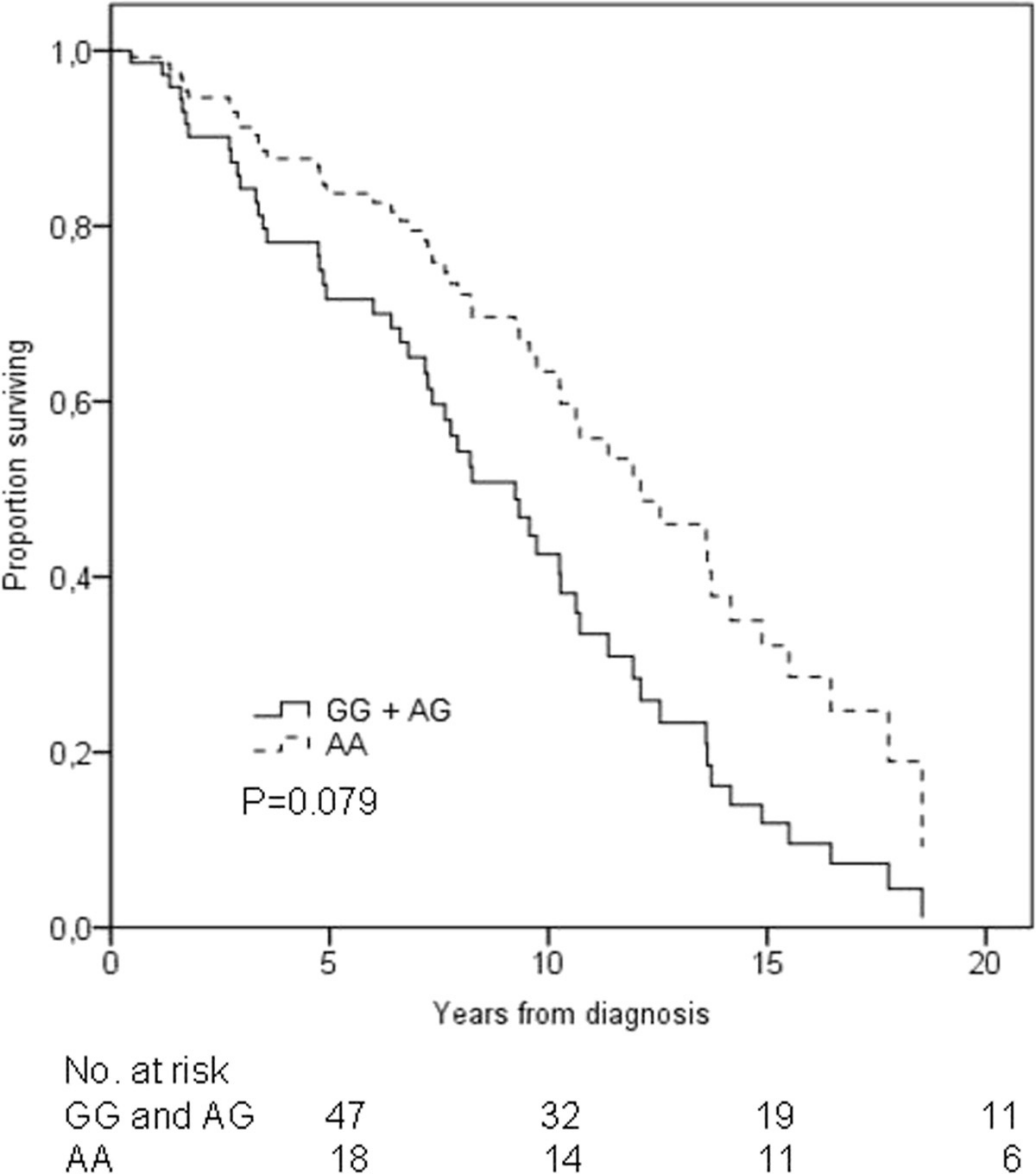
***SULT1A1 *rs9282861 genotype and overall survival of breast cancer patients receiving adjuvant tamoxifen**. The black solid line represents patients homozygous or heterozygous for the rs9282861 G allele and the black dotted line represents patients homozygous for the rs9282861 A allele. Adjustments were made for stage, age, and radiation therapy.

In the multivariate analysis adjusting for age, stage and adjuvant radiation therapy, the OS did not differ significantly between the patients homozygous for the variant A allele and the patients carrying the wild-type G allele (HR = 0.53, 95% CI = 0.27-1.08, *P *= 0.079) (Figure 2, Table 2). In terms of BCSS or RFS, there were no statistically significant differences according to the rs9282861 genotype. The Kaplan-Meier curves for BCSS are shown in the Additional file 3: Figure S2.

### Influence of the rs9282861 SNP on survival of the combined patient population receiving adjuvant TAM or chemotherapy

**Figure 3:**
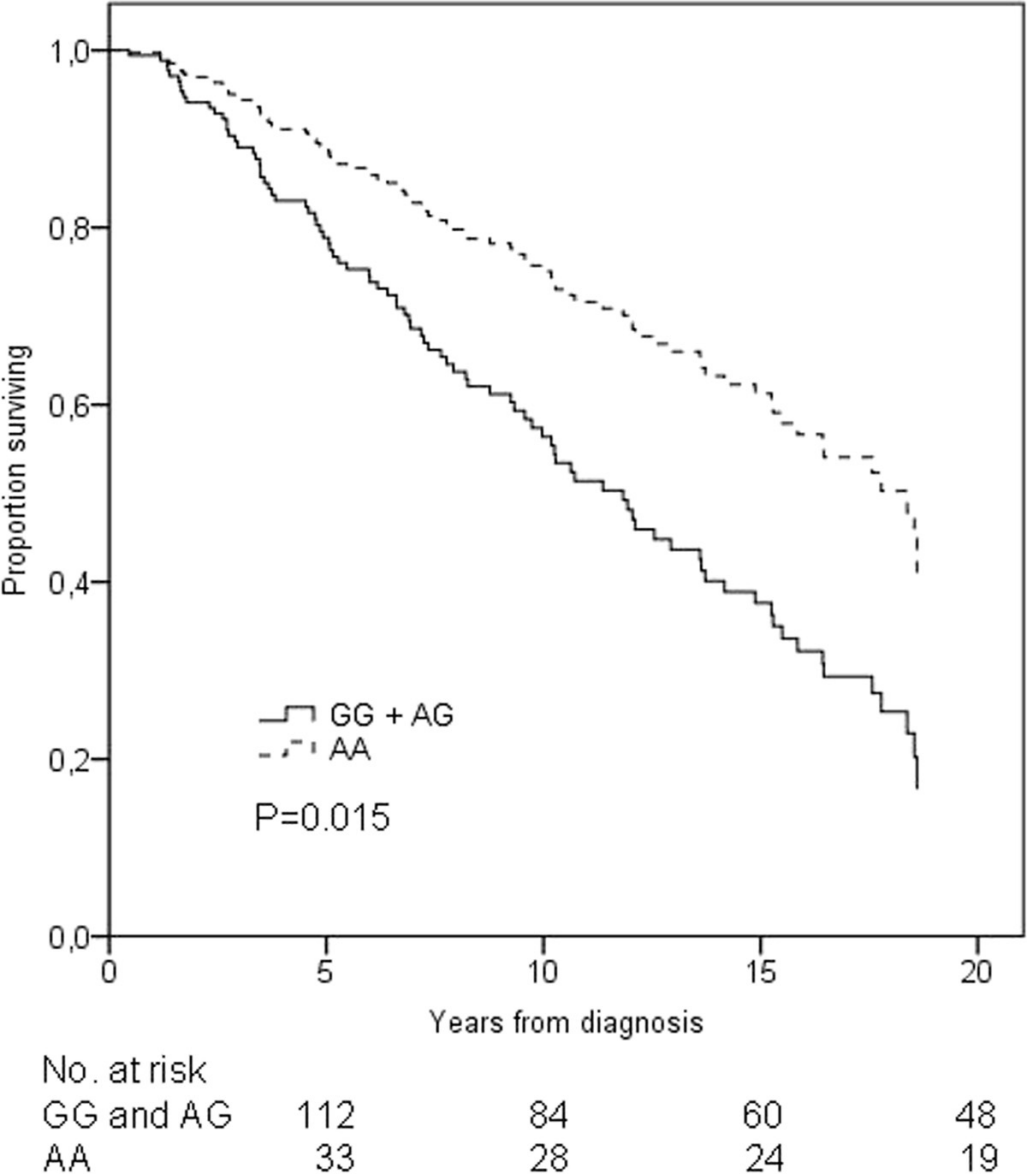
**Overall survival of combined patient population receiving adjuvant tamoxifen or chemotherapy according to the *SULT1A1 *rs9282861 genotype**. The black solid line represents patients homozygous or heterozygous for the rs9282861 G allele and the black dotted line represents patients homozygous for the rs9282861 A allele. Adjustments were made for stage, age, radiation therapy, and hormone receptor status.

Altogether 141 patients received either chemotherapy or TAM as their adjuvant treatment. In addition, four patients were given both chemotherapy and TAM. The univariate analysis of these 145 patients detected a significant difference in OS (log-rank *P *= 0.042). The BCSS did not differ significantly (log-rank *P *= 0.088) (Additional file 4: Figure S3). After adjusting for age, stage, adjuvant radiation therapy, and hormone receptor status, the multivariate analysis showed that patients with the homozygous variant rs9282861 AA genotype had statistically significantly improved OS (HR = 0.50, 95% CI = 0.29-0.88, *P *= 0.015) (Figure 3, Table 2). A parallel although statistically insignificant pattern was seen in BCSS (HR = 0.53, 95% CI = 0.26-1.05, *P *= 0.069). No statistically significant difference was seen in the RFS (*P *= 0.091).

In the dominant model there were no statistically significant differences in survival in any of the treatment groups. In contrast to the adjuvant chemotherapy or TAM treated patients the *SULT1A1 *rs9282861 SNP did not have any influence on the survival of patients not receiving medical adjuvant therapy. This explains why the rs9282861 genotypes did not appear as a prognostic factor in the survival analyses for the whole study population (n = 412).

## Discussion

The aim of this study was to determine whether the *SULT1A1 *rs9282861 genotype is associated with clinical outcome of patients diagnosed with early breast cancer and treated with either adjuvant TAM or chemotherapy. Our study had a median follow-up of nearly 12 years and it provides data on overall, breast cancer specific and relapse-free survival. The multivariate analysis of the combined patient population given either TAM or chemotherapy showed a statistically significant association between the studied rs9282861 SNP and OS, favouring patients with the homozygous variant AA genotype. However, in a separate analysis of patients receiving either adjuvant chemotherapy or TAM, the differences in survival were not statistically significant.

Our finding of improved survival of patients homozygous for the variant *SULT1A1 *rs9282861 A allele is in agreement with the hypothesis that the lower catalytic activity associated with the homozygyous AA variant genotype might lead to slower elimination of 4-OH-TAM, thus lengthening its duration of action. On the other hand, based on our results rs9282861 genotype is not a distinct predictive factor for the efficacy of adjuvant TAM or chemotherapy since BCSS did not differ significantly. As we analyzed all the 412 patients, including those who were given only adjuvant radiotherapy and those who did not receive any type of adjuvant treatment, there was no difference in OS or BCSS. Therefore, the rs9282861 genotype did not seem to be an independent prognostic factor in our unselected breast cancer patient population. Instead, the rs9282861 genotype emerged as a statistically significant prognostic factor as we analyzed OS specifically for the patients given medical adjuvant treatment.

However, our finding is not supported by previous clinical studies [8,9,11,26,27]. To explain the improved OS of the carriers of the wild-type G allele, Nowell et al. [8] suggested in their study of TAM treated breast cancer patients (n = 160) that the sulfated form of 4-OH-TAM is reabsorbed in the kidney and further desulfated in the breast tumours by steroid sulfatase, thus prolonging the duration of action of the active 4-OH-TAM. Another possible explanation was that the high-activity allele induces global expression of the SULT1A1 enzyme, followed by increased elimination of potentially harmful substrates.

In another study with a similar follow-up time as our study but with a slightly different approach, Wegman et al. [11] investigated the influence of the *SULT1A1 *rs9282861 genotype on RFS of breast cancer patients treated with adjuvant TAM or no endocrine therapy. In the group of TAM-treated patients (n = 112) there was a trend of lower risk of distant recurrence among carriers of the wild-type GG genotype. It is noteworthy that in their study genotyping was made from tumour tissue, which may cause a risk of genotype misclassification. However, the most plausible reason for the discordant results between different studies is the lack of power due to small sample sizes.

The outcome of TAM therapy is probably not solely determined by a single SNP but a combination of several genetic factors. In addition to sulfation by SULTs, glucuronidation of TAM and its metabolites by uridine diphosphate glucuronosyltransferases (UGTs) offers a way of substrate elimination through the bile. Glucuronidation is probably the most effective way to excrete TAM and its derivatives [28]. In fact, the *UGT2B15 *high activity genotype has been associated with an increased risk of recurrence and poorer survival in a group of TAM-treated patients [26]. Furthermore, several other UGTs (UGT1A4, UGT2B7, UGT1A8 and UGT1A10) have been reported to be active against 4-OH-TAM [29,30].

Polymorphisms associated with the CYP genes, especially *CYP2D6*, may also have a substantial effect on the outcome of TAM therapy; CYP2D6 contributes to the formation of 4-OH-TAM in human liver [31]. Moreover, TAM is metabolized to 4-desmethyl-TAM via CYP-dependent pathway by CYP3A4 and secondarily to endoxifen by CYP2D6, and decreased CYP2D6 enzyme activity has been associated with worse event-free survival and disease-free survival in patients treated with adjuvant TAM [32], although contradictory results have also been reported [11]. This complexity of TAM metabolism may explain the conflicting results in different studies.

There appears to be no studies on the role of *SULT1A1 *polymorphism in the pharmacokinetics of chemotherapeutic regimens, and the mechanism of this potential association is unclear. It is known that heterocyclic amines are activated by SULTs [33]. The sulfonate group is often transferred to oxygen, which is frequently in the form of hydroxyl group [33]. In theory, 4-OHCPA might serve as a substrate to SULT1A1 and possessing the high-activity *SULT1A1 *allele would increase the rate of elimination of CPA, thus decreasing the individual's exposure to its cytotoxic effects. However, none of the chemotherapeutic drugs given in the CMF regimen are known to act as substrates to SULT1A1. In addition, to date there are no pharmacokinetic or in vitro data available to support this hypothesis.

Our results clearly indicate that there may be an association between the *SULT1A1 *rs9292861 genotype and the survival of breast cancer patients, but further studies are warranted due to a relatively small sample size. Lack of specific data on the other medications used by the patients is also a limitation of our study. For example, concomitant use of CYP2D6 inhibitors, including selective serotonin reuptake inhibitor (SSRI) antidepressants, may reduce the efficacy of TAM [34]. However, the influence of this potential confounding factor is anticipated to be minor since the use of SSRIs was uncommon in the 1990s.

Lastly, local radiotherapy was given to 77 patients (95.1%) receiving chemotherapy and to 47 patients (72.3%) treated with TAM. In the univariate analysis, the rs9282861 genotype was not associated with any differences in survival among patients who were given adjuvant radiotherapy but no adjuvant chemotherapy or hormonal therapy (n = 230). It is unlikely that radiotherapy interacts with SULT1A1 enzyme, which would result in various survival outcomes between *SULT1A1 *genotypes.

## Conclusions

In summary, breast cancer patients with the *SULT1A1 *rs9282861 homozygous variant AA genotype and treated with either adjuvant TAM or chemotherapy had statistically significantly better OS compared with the carriers of other rs9282861 genotypes. However, the association was not statistically significant in the multivariate analysis conducted among patients given only chemotherapy or TAM. Moreover, the BCSS did not differ significantly between the carriers of different rs9282861 genotypes. Further prospective studies with larger samples are therefore needed to assess the real relevance of the present findings and their potential influence on treatment outcomes of breast cancer patients.

## Competing interests

The authors declare that they have no competing interests.

## Authors' contributions

MT participated in the data collection and interpretation and statistical analysis and drafted the manuscript. AH, VK, VMK and AM read the draft and critically revised the manuscript. AH was responsible for genotyping analyses. VMK participated in study design, data collection and interpretation. AM participated in the statistical analysis and data interpretation. VK participated in the study design, data collection and interpretation. All authors have read and approved the final version of the manuscript.

## Pre-publication history

The pre-publication history for this paper can be accessed here:

http://www.biomedcentral.com/1471-2407/12/257/prepub

## Supplementary Material

Additional file 1 **Table S1**. Causes of death according to the *SULT1A1 *rs9282861 genotype in the cohort of patients treated with adjuvant chemotherapy (n = 76). **Table S2**. Causes of death according to the *SULT1A1 *rs9282861 genotype in the cohort of patients receiving adjuvant tamoxifen (n = 65). **Table S3**. Causes of death according to the *SULT1A1 *rs9282861 genotype in the combined patient population receiving adjuvant tamoxifen or chemotherapy (n = 145). Click here for file

Additional file 2 **Figure S1**. Kaplan-Meier survival curves for BCSS according to the *SULT1A1 *rs9282861 genotype in the cohort of patients treated with adjuvant chemotherapy. Click here for file

Additional file 3 **Figure S2**. Kaplan-Meier survival curves for BCSS according to the *SULT1A1 *rs9282861 genotype in the cohort of patients treated with adjuvant tamoxifen. Click here for file

Additional file 4 **Figure S3**. Kaplan-Meier survival curves for BCSS according to the *SULT1A1 *rs9282861 genotype in the combined patient population receiving adjuvant tamoxifen or chemotherapy. Click here for file
