# Supplementary figures and images for: SULT1A1 rs9282861 polymorphism-a potential modifier of efficacy of the systemic adjuvant therapy in breast cancer?
Source: BMC Cancer. 2012 Jun 18;12:257. doi: 10.1186/1471-2407-12-257 (PMC3388009; doi:10.1186/1471-2407-12-257)

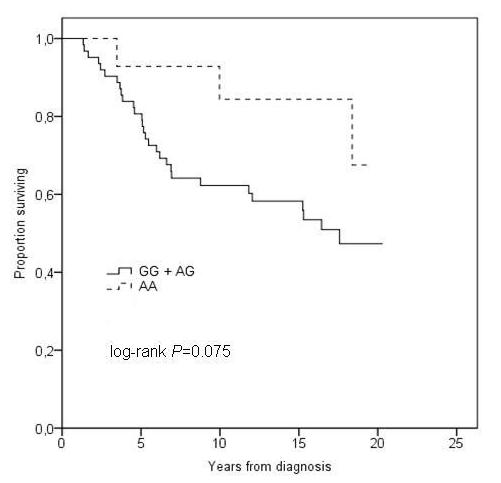

Supplement: Additional file 2 — Figure S1. Kaplan-Meier survival curves for BCSS according to the SULT1A1 rs9282861 genotype in the cohort of patients treated with adjuvant chemotherapy. [file 1471-2407-12-257-S2.JPEG]

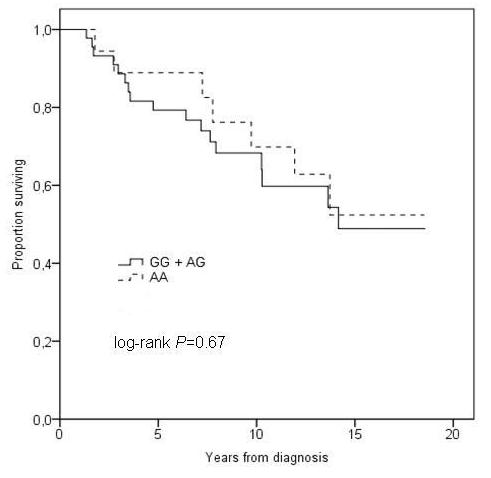

Supplement: Additional file 3 — Figure S2. Kaplan-Meier survival curves for BCSS according to the SULT1A1 rs9282861 genotype in the cohort of patients treated with adjuvant tamoxifen. [file 1471-2407-12-257-S3.JPEG]

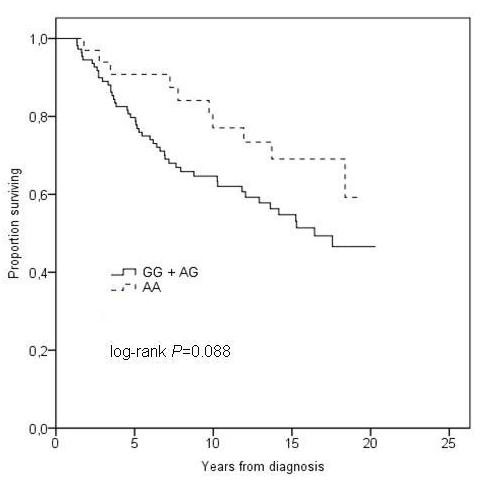

Supplement: Additional file 4 — Figure S3. Kaplan-Meier survival curves for BCSS according to the SULT1A1 rs9282861 genotype in the combined patient population receiving adjuvant tamoxifen or chemotherapy. [file 1471-2407-12-257-S4.JPEG]
